# A digital physician peer to automatically detect erroneous prescriptions in radiotherapy

**DOI:** 10.1038/s41746-022-00703-9

**Published:** 2022-10-21

**Authors:** Qiongge Li, Jean Wright, Russell Hales, Ranh Voong, Todd McNutt

**Affiliations:** 1 Department of Radiation Oncology and Molecular Radiation Sciences, Johns Hopkins University School of Medicine, Baltimore, MD 21205 USA; 2 Present Address: Department of Radiation Oncology, Brown University, Providence, RI 02912 USA; 3 Present Address: Department of Radiation Oncology, Rhode Island Hospital, Providence, RI 02903 USA

**Keywords:** Software, Information technology

## Abstract

Appropriate dosing of radiation is crucial to patient safety in radiotherapy. Current quality assurance depends heavily on a physician peer-review process, which includes a review of the treatment plan’s dose and fractionation. Potentially, physicians may not identify errors during this manual peer review due to time constraints and caseload. A novel prescription anomaly detection algorithm is designed that utilizes historical data from the past to predict anomalous cases. Such a tool can serve as an electronic peer who will assist the peer-review process providing extra safety to the patients. In our primary model, we create two dissimilarity metrics, *R* and *F*. *R* defining how far a new patient’s prescription is from historical prescriptions. *F* represents how far away a patient’s feature set is from that of the group with an identical or similar prescription. We flag prescription if either metric is greater than specific optimized cut-off values. We use thoracic cancer patients (*n* = 2504) as an example and extracted seven features. Our testing set *f1* score is between 73%-94% for different treatment technique groups. We also independently validate our results by conducting a mock peer review with three thoracic specialists. Our model has a lower type II error rate compared to the manual peer-review by physicians.

## Introduction

Radiotherapy (RT) is a complex process that requires careful quality assurance to ensure safe treatment delivery. One common safety concern is with errant or uncommon prescriptions inadvertently being administered: excessively irradiating the patient can lead to injury or death. Meanwhile, under-irradiating may fail to mitigate cancer. Even though such events are rare, the impact of missing such errors could be catastrophic, and minor deviations result in sub-optimal treatment.

Peer review (PR) chart rounds are a significant component of the current quality assurance program in radiation oncology departments. PR chart rounds are a requirement of the American Society for Radiation Oncology, American College of Radiology, and the American Association of Physicists in Medicine^1^. However, a recent study^2^ highlighted that PR is not a perfect system, especially when it is conducted late in the patient care life-cycle, and that there remains room for improvement. In a study intended to evaluate the effectiveness of the PR process^3^, erroneous prescriptions and other anomalous cases were inserted into weekly rounds over nine weeks. Only 67% of these anomalous prescriptions were detected by the physicians. Our goal is to present a data-driven algorithm to assist physicians by detecting anomalies automatically, which could potentially improve the patients’ safety.

There is an increasing trend to study how machine learning (ML) tools can be used to augment medical professionals’ decisions concerning diagnosis, treatment safety, and quality of patient care^4–10^. Several pharmaceutical studies^11–15^ have applied ML to find anomalous prescriptions but not tailored to RT. In RT, several studies^16–19^ have used ML to look at the treatment parameters to detect errors in treatment plans, but did not focus on prescription error detection.

This work presents a multi-layer prescription anomaly detection tool that creates an automated, historical data-driven checkpoint to assist in PR. The tool’s core utilizes a ‘distance model’, which defines distance metrics between a new patient’s features and prescriptions and those in a historical database. Prescription elements are the dose per fraction and the number of fractions prescribed to the target volume. Besides prescription features, there are other features such as diagnosis code, age at treatment, disease stage, treatment intent. Using a logical rule-based approach, the model will flag the new patient’s prescription as anomalous if the distances fall outside certain optimized thresholds within a subgroup of similar patients.

## Results

### Distance model results

Here, we provide illustrative results from running the distance model. In Supplementary Figure 3, we plotted the histograms of prescription and feature distances from the historical database. We can see that the prescription distances of zero or 0.2 are particularly common, which reflects the fact that many patients in the dataset have the same or similar prescriptions. The feature distances are more varied, and display characteristic spikes associated with the categorical differences (see Supplementary Figure 3 caption for further explanation).

As discussed in the Methods, there are several ways in which we synthesized anomalies. We present all results in Table 1. The *S* column refers to the number of records in the historical database, *a*,*b* are the parameters multiplying *θ* and *τ* respectively, and $\mu =\frac{m}{S}$ and $\nu =\frac{n}{S}$ are the parameters *m* and *n* expressed as percentages of *S*. *s*_*a*_ refers to the number of anomalies in the training or test set, whereas *s*_*n*_ refers to the number of normal *holdout* historical samples in the training or test set. Note that the holdout set *s*_*n*_ is not used to compute *θ* or *τ*.

**Table 1:** Parameters and model performance scores.

	Technique	a	b	*ν*	*μ*	*τ*	*θ*	*f*1	*s*_*n*_	*s*_*a*_	*S*
Rx switched SAs	3D	0.449	1.632	0.012	0.018	0.581	0.206	0.98 ± 0.03	20	10	509
	IMRT	0.265	0.979	0.025	0.014	0.543	0.261	0.89 ± 0.01	20	10	1153
	SBRT	1.631	1.838	0.047	0.014	0.501	0.142	0.98 ± 0.03	20	10	704
Feature switched SAs	3D	0.056	0.797	0.021	0.019	0.581	0.206	0.84 ± 0.02	20	20	509
	IMRT	0.286	0.802	0.023	0.038	0.543	0.261	0.84 ± 0.01	20	20	1153
	SBRT	0.307	0.584	0.017	0.029	0.501	0.142	0.90 ± 0.03	20	20	704
In-sample (both types of SAs)	3D	0.010	0.717	0.010	0.037	0.581	0.206	0.84 ± 0.01	30	30	499
	IMRT	1.401	0.805	0.025	0.014	0.543	0.261	0.86 ± 0.01	30	30	1143
	SBRT	1.926	0.465	0.01	0.075	0.501	0.142	0.91 ± 0.03	30	30	694
Out-of-sample (both types of SAs)	3D	0.010	0.717	0.010	0.037	0.580	0.200	0.941	10	8	529
	IMRT	1.401	0.805	0.025	0.014	0.544	0.273	0.727	10	10	1173
	SBRT	1.926	0.465	0.010	0.075	0.503	0.141	0.875	10	7	724

For in-sample, the *f*1 score was computed by averaging over 50 trials of random samples of the not-anomaly holdout set *s*_*n*_. We found *f*1 scores of 0.98 for 3D, 0.89 for IMRT, and 0.98 for SBRT, where the error bars run between 2–5%. For the feature switching generated SAs, we found *f*1 scores of 0.84 for 3D, 0.84 for IMRT, and 0.90 for SBRT with similar error bars, as shown in Table 1.

Next, we ran the model on a training set combining both prescription-switched and feature-switched SAs. We found that the resulting *f*1 scores for the combined training set lie in between the scores for the training sets where each type of anomaly was considered separately. This makes sense intuitively. We report the results and parameters in Table 1. Because the standard deviation is small, we choose any run as our final parameters. Note that *τ* or *θ* varies slightly because of the different historical holdout samples.

Out-of-sample results are obtained by running the distance model with the same parameters that were found during optimization over the training set, on the new unseen test set. E.g., in the test set, both the normal ‘non-anomalous’ test records and the anomalous test records are previously unknown to the distance model.

We used a separate, recent data set (01/01/2021 - 07/14/2021) to select samples for our out-of-sample testing nonanomalous class data. We used all of the samples during this time period for the 3D and SBRT, each containing ten samples. We selected 10 of the most typical cases out of the 24 IMRT samples from this time period as our testing normal class. For the out-sample case, the historical data set (from 01/01/2006 - 12/31/2021) is still an important input into the model, however, no samples are drawn from it for prediction. We then created a new set of SAs for each technique using several construction methods and verified the anomalous class status by looking at the conditional feature distribution after switching/changing features.

We report the out-of-sample distance model results in Table 1. We can see that comparing the out-of-sample performance to the in-sample, the out-of-sample is worse for IMRT and SBRT but better for 3D.

A beneficial feature of the distance model is that not only do we get the model prediction for each of the test records, but we also get an explanation of why each prediction was made. By looking at the values of *R*, *F*, *t*_*F*_ and *t*_*Rx*_ we can immediately see the reason why a sample was flagged or not flagged, as shown for example in Table 2, where each row represents a testing patient.

**Table 2:** Prediction examples.

Fx	Dose/Fx(*cGy*)	Tech	Energy(MeV)	Intent	ICD10	ICDO	Age	Truth	Pred	Type	R	*t*_*Rx*_	F	*t*_*f*_	Counts
4	1200	SBRT	10fff	palliative	C15.6	87203	49	1	1	2	0.00	0.27	0.56	0.23	417
4	1200	SBRT	6fff	curative	C34.12		61	0	0		0.00	0.27	0.21	0.23	417
4	500	3D	mixed photon		C34.90	80463	76	1	1	1	0.13	0.00			1

The ‘Truth’ column refers to whether the data point is actually an anomaly or not (1 indicates anomaly; 0 indicates normal). The ‘Pred’ column is the prediction by the model, where again 1 indicates anomaly and 0 indicates not. The first row was predicted by the model to be a ‘type II’ anomaly, which means the feature distance is large for this patient compared to the historical database (the new patient’s feature sets do not match well with the population who received the same prescription in the past). *R* is 0, which is below the cut off *t*_*Rx*_ but *F* is larger than the cut off value *t*_*F*_. *R* is zero because this prescription has been seen in the historical database (The ‘Counts’ column indicates it has been seen 417 times previously). Observation of historical distributions shows that the energy 10fff was never previously used for the prescription 4 *fx* x 1200 *cGy*, and this prescription was never used to treat an esophagus diagnosis either. This, again, shows that being a “common” prescription cannot promise being “normal” or not an error.

The second row is a normal patient in the database where the feature sets match well with the historical record. Therefore *R* and *F* are both smaller than cut-off values. In the third row, we show a switching anomaly, the original prescription was 5 *fx* x 400 *cGy*, but we switched it to 4 *fx* x 500 *cGy*. This leads to a large prescription distance *R*, making the model predict it as prescription anomaly (‘type I’). This is consistent because 4 *fx* x 500 *cGy* almost never appears in the historical database (Counts = 1). This again, shows that our model has the ability to not only predict anomalies but also to *explain* each prediction.

### Mock peer review (PR) results

**Fig. 1:**
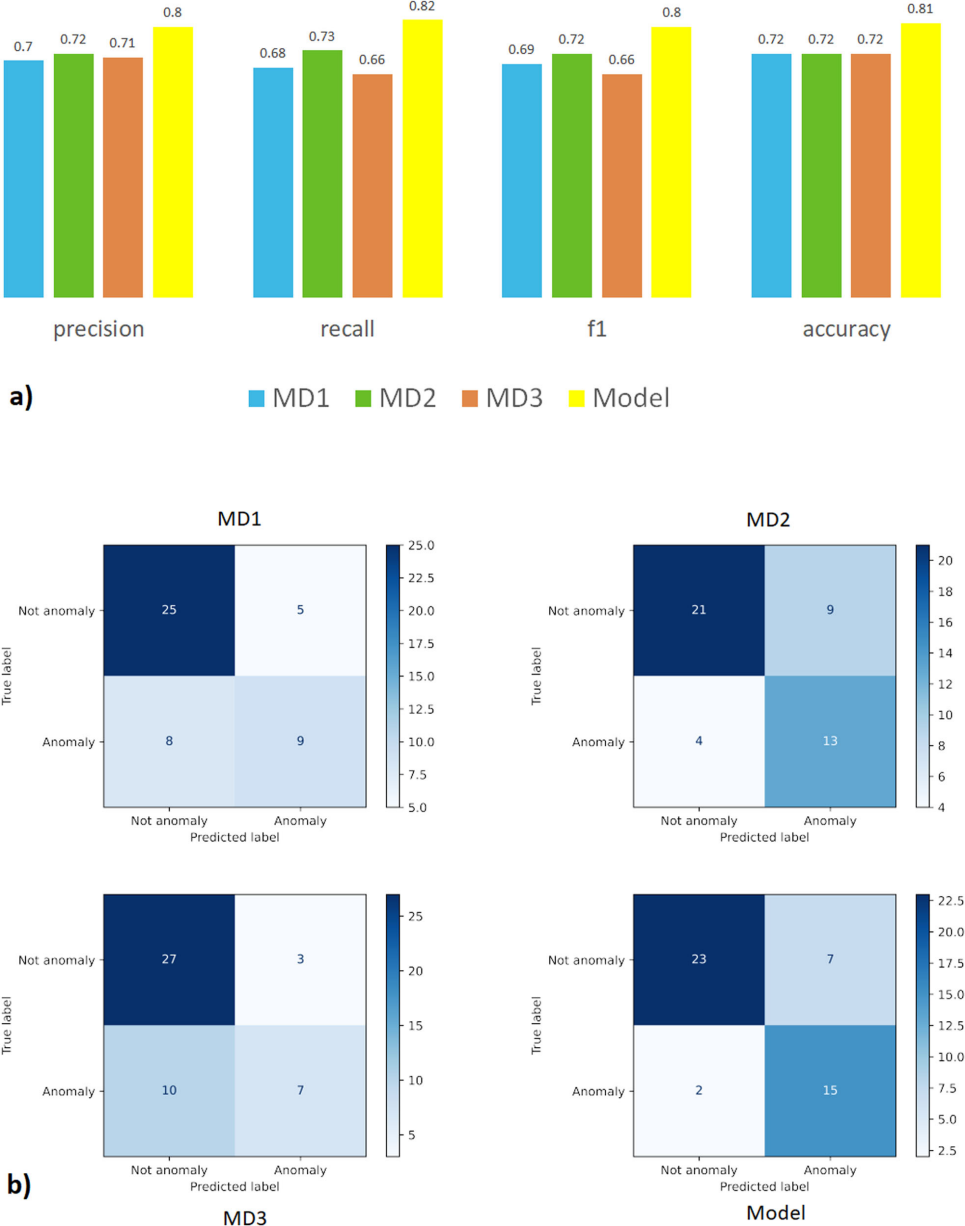
Performance. **a** Performance (macro average of metrics) of physicians vs. model. The blue, green and orange bar indicates each physician’s performance and the yellow bar is the model performance. We can see that the model’s precision, recall, f1 and accuracy scores are all compatible with the physicians suggesting that the model can serve a role as a digital peer. **b** Confusion matrix. The model has the lowest false-negative rate, suggesting that the model is more conservative than all the physicians, in deciding whether a case should be considered an anomaly.

In order to independently validate our results, we conducted a mock PR. Three radiation oncologists with more than ten years of experience treating thoracic patients were each asked to independently label a sample dataset containing 17 anomalies and 30 normals (a subset randomly selected from out-of-sample testing data). The results of the physicians, side-by-side with the model results, are shown in Fig. 1a). The performance was evaluated by calculating precision, recall, f1 and accuracy. Additionally, confusion matrices for the physicians (MDs) and the model are shown in Fig. 1b) which gives a breakdown of the different type I and type II errors made by each physician and the model. We can see that the model slightly outperformed each physician at the individual level. In Supplementary Table 6, we show some specific examples of cases and how the MDs performed compared to the distance model.

### Time analysis

To get a sense of the time and effort spent by each physician on the mock PR, we asked each physician to note the time spent on the review. MD2 spent 18 min identifying the errors and 12 min writing out the rationale. MD1 spent a total of 11 min both identifying the errors and writing out the rationale for their decisions. The model running time for a single testing sample is about 1s and the model training time is several days. However, one only needs to train the model once for a given historical database (until there is a major update of new historical data). The training time is proportional to the number of evaluation points in the grid space, the number of runs to average the *f*1 score and the number of data samples.

### Model’s performance vs. physician group’s performance

In the PR, physicians can discuss each case and combine their knowledge to form a consensus about the correctness of a prescription for each case under review. Thus, besides comparing our model’s performance against each physician individually, we also compare it with the group consensus. We consider a best and worst-case scenario from joining MDs. In the best case, the consensus is correct if any MD was correct; in the worst case, the PR selects an incorrect decision if any MD was wrong. We would expect actual performance of PR in the real clinical setting would lie in between.

**Fig. 2:**
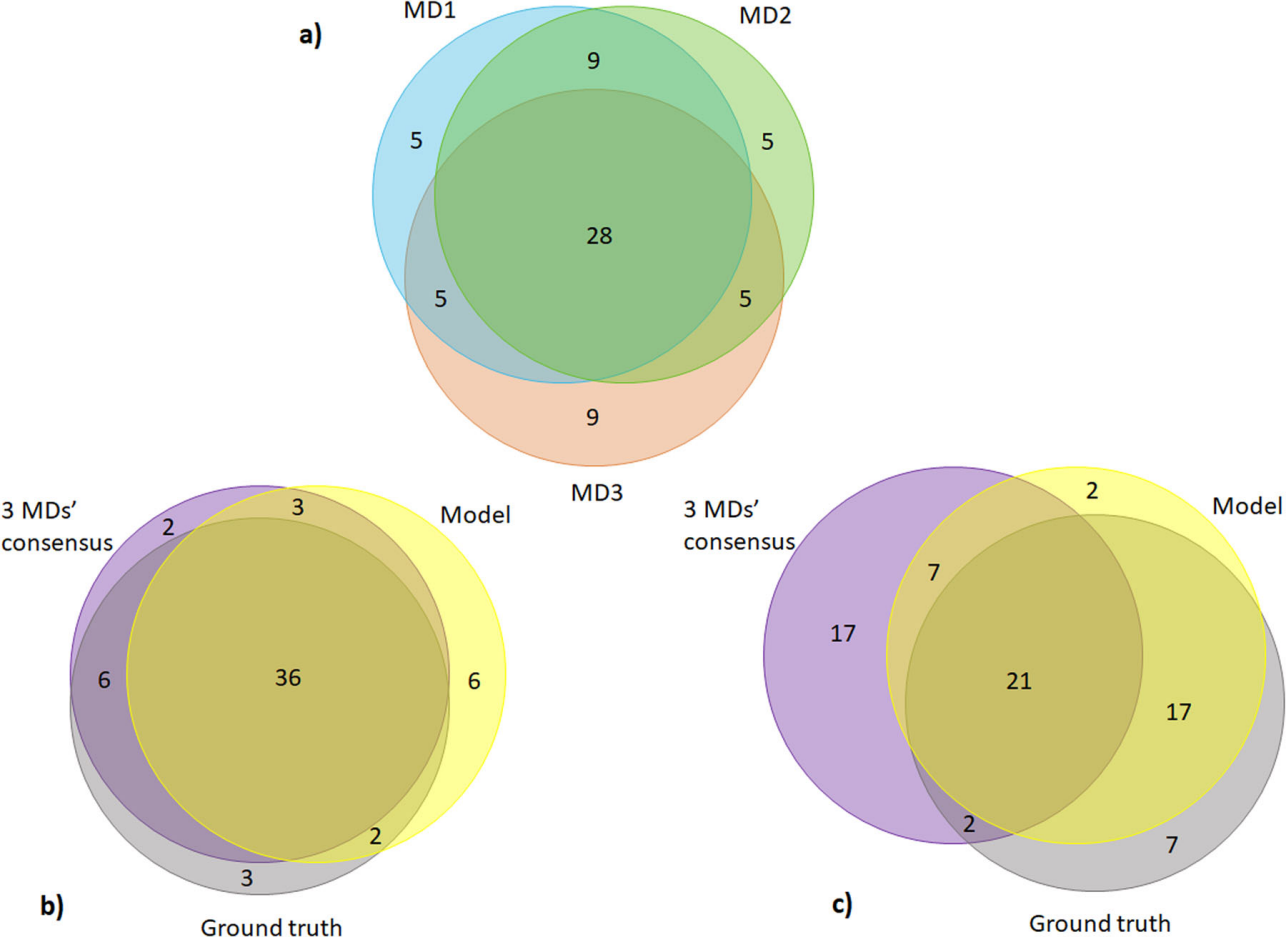
Overlap of agreement between MDs, the model and the ground truth. Panel **a** shows the overlap of agreement between the three MDs on decisions of whether to flag or not to flag a particular case. Panel **b** shows the best-case scenario from the PR and **c** shows the worst-case scenario.

The results of such a worst and best-case scenario are displayed in Fig. 2 as well as the overlap diagrams of agreement for each individual MDs. Note that the numbers in the Venn diagrams do not distinguish between anomalous or non-anomalous class. Any overlap regions with the ground truth set correspond to correct decisions, any decisions outside the ground truth set correspond to incorrect decisions. We can see that, in the worst case, as shown in panel c), the model outperformed the consensus by missing 9 cases rather than 24 cases by the consensus.

However, the real question is whether the model is still better than the best-case consensus, as shown in panel b)? The answer is no. The model missed 9 cases, while the best-case consensus missed 5 cases. Our model’s performance is in between the best and worst scenario, but closer to the former. The overlapping regions/agreements indicates that the model independently agreed with physician’s knowledge.

We should not interpret these results to suggest that the model under-performed or out-performed the MDs in the mock PR. Instead, we suggest that the model be considered an additional “digital peer reviewer” to complement the MDs. Under these circumstances, the distance model has promise as a validation tool to check for prescription errors since the model caught anomalies that the physicians overlooked.

## Discussion

It is important to note that while the intent of the model is to detect erroneous prescriptions, there will, nevertheless, be cases where the flagged prescription is rare but not erroneous. Such instances are false positives (wrongly flagged) by the model. However, it makes sense to flag prescriptions that are rare, as well as prescriptions that are erroneous, as both cases warrant further scrutiny from the peer review team.

An important underlying working assumption of our model is that our final historical database that is fed into the distance-model component is error-free. In the Methods, we described how we manually inspected the historical database and attempted to clean it of anomalies. Even with this step taken, the assumption may not hold exactly true and whatever erroneous data points that lie undetected in our historical database will cause some error rate in our model. The averaging parameters *m*, *n* were introduced into the model for this very reason in order to reduce the potentially harmful effects of a single error in the historical database.

Therapeutic radiation oncology is a fast-evolving field where the clinical practice were influenced by the practice guidelines and protocols. This is one of our motivations for choosing a data-driven approach because we can evolve the models with the data instead of having to constantly keep up with standard-of-care protocols. We fully appreciate the changes in practice over time and have published on it before^20^. The anomaly detection is a learning tool that highlights aberrant patterns. When new techniques and combinations are added to clinical practice, we anticipate a transient increase in detection associated with these changes. These false-positive detection results are significant because the patients treated at the time of an evolving standard of care may need extra caution taken in treatment plan review. As the clinical team takes additional time for judgment on these ‘detected’ cases, the tool is self-learning such that over time, the progressive changes will blend into clinical practice, no longer be anomalies in our data. Consequently, the tool will self-correct over time to keep pace with evolving standards of practice.

For example, as shown in Supplementary Figure 4, our data shows there is trend of adapting hypofractionations (300 *cGy*, 350 *cGy* and 400 *cGy* in 15 fractions) in treating lung cancer patients since year of 2016. This evidenced that our data is reflecting the influencing study published in September 2015^21^. If we were using this tool in 2016, these new prescriptions would initially be flagged first and thereby generate discussion (appropriately), and then, as they become more common, they would eventually not be flagged any more.

However, while the data-driven aspect may be useful in some cases, it would be naive to think that we can allow the model to be generally indifferent to secular (time) trends in RT prescription guidelines. It might be the case that a prescription (treatment guideline) was very popular in the past but has become outdated by today’s standards. This could lead our model to fail to reach the right decision. For this reason, we conducted an analysis to systematically search for secular trends in the historical database, checking every prescription. The results of this analysis are presented in Supplementary Notes. We find that for our dataset, several prescriptions display statistically significant time trend behavior and propose a methodology for including this information into our pipeline to correct our predictions in the presence of secular trends.

In machine learning binary classification, a common problem is that there are far more instances of the majority class than the minority class in the training set (anomaly detection is the prime example of this). Consequently, the model training tends to ignore the minority class unless the model complexity is allowed to grow into the high variance region. One of the advantages of the distance model compared to a supervised learning (SL) model is that it does not present any problem with class imbalance. This is because the distance model is not a SL model in the traditional sense and instead relies on distances between historical data and the test set to define outcomes. When comparing the model’s performance versus the physician’s performance we note that even with the same level of performance, the model is still valuable because it is a fully automated process that does not require valuable physician time and provides an additional safety check.

Rajkomar et al. ^4^ mentioned that rule model-based methods are preferred over traditional machine learning algorithms when the problem is relatively straightforward with limited but informative variables – this is our case. With respect to traditional supervised and unsupervised learning methods, it is possible that similar results could be constructed with existing anomaly detection techniques such as isolation forest, local outlier factor or some adaptation of *k*-means. However, it is unclear how to separate the prescription features from the diagnostic/other features, as discussed in the Introduction.

Recent work in the field of patient similarity learning (PSL) has introduced a number of novel methodologies to stratify patients into sub-groups^22^. Of particular relevance to radiotherapy are the strategies that integrate clinical data with imaging data. With cancer as the underlying clinical domain, genetic data is also relevant to the patient similarity metric if it can be included. Any similarity metric can be used for anomaly detection by looking for large values of the metric or patients that belong to certain sub-groups.

For example, Li et al.^23^ developed a network topology-based method to define sub-groups of similar patients with type II diabetes. This is a general method that can be used with any dataset that has electronic medical records and genotyping single nucleotide variations. In reference^24^, a similarity metric was developed based on RT image data, it would be interesting to see if we can integrate our image data into our pipeline with a similar system. There are also a number of visualization software such the ICM^25^, which allows for clustering and visualization of high-dimensional biomedical data. Such a tool could be used to visually identify anomalies outside clusters, which is an interesting alternative anomaly detection method.

These methods can be applied to our problem of anomaly detection to better define patient sub-groups and optimal treatment patterns. We would need to expand our data types to include imaging or genetic data alongside the clinical features. Finding the right level of granularity in sub-grouping patients is a challenge^22^. In our study we made no attempt to sub-group patients and treated them all at the same level (though we do introduce the *m*-closest and *n*-closest subset of most similar historical patients using our straightforward metrics). More common approaches to patient stratification in PSL include *k*-means or hierarchical clustering as well as PCA for dimensionality reduction to try to simplify the features for sub-grouping. Including *k*-means, for example, as an intermediate grouping step in our pipeline may improve the overall ability of the pipeline to identify appropriate/anomalous treatment prescriptions.

Another alternative method in this context^26^ calculates the conditional probability of the prescription conditioned on the features and threshold for the rarity. We could have expanded this idea by calculating every conditional probability of the features on the prescription, or features on other features and threshold for rarity for the same prescription. However, a major drawback of this approach is that it involves many condition-by-condition checking of histograms. In contrast, our approach is simpler where we save effort in avoiding checking case-by-case.

However, we are limited by the number of informative features that we can build and the available data. Lack of features limits our ability to make predictions, and lack of data increases the variance in whatever predictions we can make.

To increase the number of features, Natural Language Processing (NLP) would be needed to encode the physician’s notes into a vector, which we can calculate pairwise distances over. More data could be obtained by merging datasets with other institutions.

Another major limitation is the difficulty of constructing or obtaining anomaly data. It is challenging to make realistic anomalies because they are rare and unexpected by their nature, so creating a set of anomalies that fully samples the space of possibilities is a significant challenge. It would be helpful to have more anomalous data for validation. The relatively small number of anomalies limits the scope of our findings.

In summary, we have provided a proof-of-concept for an anomaly detection pipeline for prescription in radiotherapy. Our results show that the distance model and connected pipeline can predict with good accuracy for anomalies that are constructed as described in the Methods. The model showed promise and was evaluated favorably in the mock clinical setting where its predictions agreed independently with physicians’ knowledge and, in some cases, out-performed the physicians. Our approach has focused on a custom decision tree rule-based anomaly detection logic that creates its own definitions of "dissimilarity” between historical patient data. These dissimilarities are incorporated into a pipeline with novel decision tree logic that is a potentially useful and novel approach to prescription anomaly detection in the RT setting.

## Methods

### Data description

Our radiation oncology-specific electronic medical record contains 14 years of cancer patients’ radiotherapy treatment records (10/07/2007- 07/13/2021). This comprises 63768 individual treatment prescriptions delivered to patients treated in the radiation oncology department of Johns Hopkins over the time span. We queried the thoracic subset of the data, excluding patients from other disease sites (prostate, brain etc.) so that our raw data contains 4951 de-identified treatment records. The initial data contained 32 fields (columns) for each record as seen in Supplementary Table 1. However, not all columns represent informative features. We extracted information related to patients’ treatment, including patient’s age at treatment, diagnosis code, morphology code, treatment intent, techniques, energy, anatomic site, tumor stages and biomarkers. Prescription data includes the number of fractions, dose per fraction, total dose, and accumulated total dose. The Institutional Review Board of Johns Hopkins University Hospital approved this.

### Preprocessing, feature engineering

Firstly, the raw data was split by technique. There were not enough samples to build models for the following treatment techniques: Intensity-modulated proton therapy (IMPT), Two-dimensional basic radiotherapy (2D), and Brachytherapy (Brachy), that we ruled out these techniques from our subsequent analysis. The techniques kept for later analysis are Three-dimensional conformal radiotherapy (3D), IMRT, and Stereotactic body radiotherapy (SBRT).

Many feature engineering steps were required to transform the columns of the data into a standardized form. Search and replace functions over the string features were implemented to collapse many alternate spellings of words into a single identifier. For example, for treatment technique, if we ignore the subtle differences, ‘rao/lpo’,‘5 field conformal’, ‘opposed laterals’, and ‘ap/pa’ can all be classified as ‘3d’. Similarly, ‘imrt ig’, ‘imrt ig abc’, ‘igvmat’, ‘imrt vmat ig abc’, ‘imrt tomo ig’, ‘tomotherapy’ were all be classified as ‘imrt’ and ‘sbrt vmat ig’,‘igsbrt’, ‘sbrt ig’ were all categorized as ‘sbrt’. In other cases irrelevant features needed to be removed. For example, Gleason scores were helpful for prostate cancer but irrelevant to the thoracic cancer.

In Supplementary Table 2, we listed the diagnosis codes for our model and confirmed the completeness and appropriateness of this list for the model. Our current tool only included thoracic patients whose primary tumor site is the lung, heart, or esophagus. We searched for re-plans and cone-down plans with their initials by finding the mismatch between the total and accumulated doses. Because they are only 2.6% of the total data points, and in order to simplify our analysis, we eliminated these patients’ re-plan treatment along with their initial treatment. We also eliminated the cone-down plan records for the same reason.

We decided to remove certain subsets of data points that were unrelated to actual patient data. For example, a number of fake patients used by medical physicists for calibration procedures exist in the database. For example, records with name fields such as “JOHN DOE” with zero total dose are not uncommon. Such records clearly are noise and are not the interesting data points for our model.

Finally, after cleaning the database of these pre-processing anomalies (see Supplementary Notes for a further discussion of these cases), we acquired 2504 rows of records for the thoracic group. Supplementary Figure 1 is a consort diagram that tracks the number of patients at each pre-processing and filtering step starting from the raw data and ending with the final input cases to the model described in the Distance model Section. Supplementary Table 4 shows a sample post-processed feature-set for a single patient.

### Model pipeline

The essential idea of the model is to compare the new patient’s prescriptions and other features to those in a historical database and to flag any suspicious patterns because they have not been previously seen or are rare. We can more precisely define the word “rare” in two ways. In the first case, the observation of the marginal empirical distribution of prescription over the entire filtered historical database of patient prescriptions provides a probability (or frequency) that each prescription has occurred without paying respect to any other patient features. In the second case, a prescription may not be “rare” in the marginal sense, however, perhaps the prescription never occurs for patients with a particular feature. For example, a certain prescription may be commonly used when the treatment intent is curative, but never (or rarely) used when the treatment intent is palliative. Thus we have two senses of the word rare, in one case the marginal, and the other, conditioned on other patients’ features.

It should be noted that the idea of the distance model is to avoid working with empirical histograms to the extent possible. Nevertheless, dissimilarity as defined by our metrics below, corresponds to rarity (low probability events in the empirical distributions). That correspondence would be difficult to make exact, formally, however, it is generally the case that the higher the value of the dissimilarity metric, the more rare the new patient’s feature set is; where the word rare is defined with respect to marginal or conditional prescription frequencies as described above.

Underlying this process is the working assumption that the historical database is error-free. The validity of this assumption is addressed in the Supplementary Notes as well as in the Discussion.

**Fig. 3:**
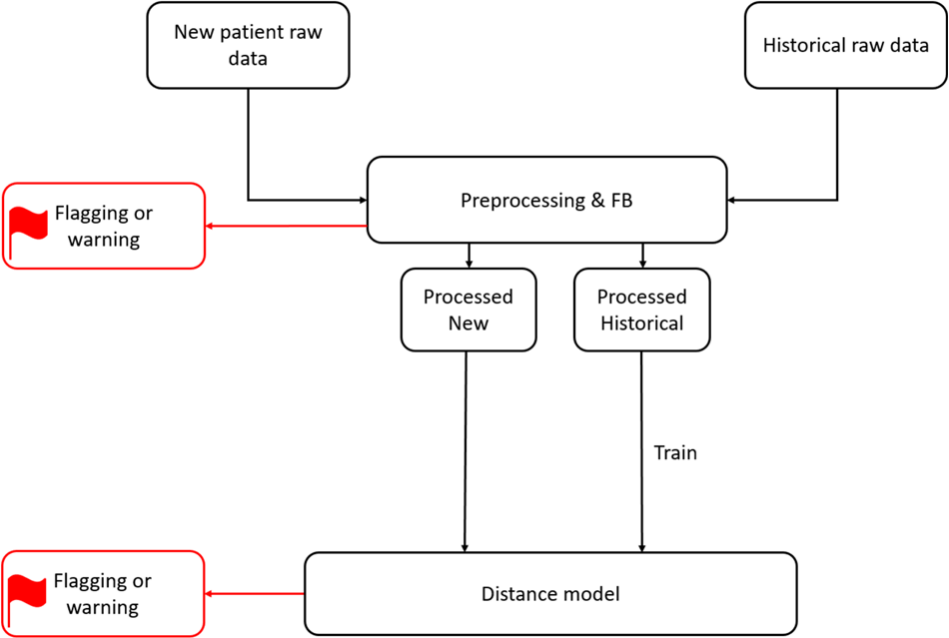
Model schematic. The flow of data through the pipeline is illustrated as well as the different pipeline components and the different places where flagging or warnings may occur.

In Fig. 3 we can see the architecture of the model. The historical data and the new patient’s are first processed as described in the previous section. Next, we explain the ‘distance model’ component of the pipeline and under what circumstances the new patient’s prescription will be flagged as a potential anomaly.

### Type I and type II anomalies

In accordance with our definition of “rare” prescription above, the distance model is designed to detect two different types of prescription anomalies. In what we call *type I* anomalies, the prescription itself is atypical from the historical records. In *type II* anomalies, the prescription is not uncommon in the historical database, however, there is a *mismatch* between the prescription and the patients’ other diagnostic features. Below we give some illustrative examples of type I and II anomalies from real clinical practice.

- A physician prescribed 5 fx × 400 cGy 3DCRT treatment for a 76-year-old Malignant neoplasm of unspecified lung patient. However, when a resident planned the case under supervision, 4 fx × 500 cGy were used. The supervising dosimetrist did not catch the error, but the physician caught the error at the time of approving the prescription. The total dose was 2000cGy in both cases, making it harder to detect the error. However, the BED was 30Gy and 20 Gy, respectively, which would cause very different radiation responses. From the historical analysis, we know that 5 fx × 400 cGy is a popular prescription that appears 98 times in the historical database, but 4 fx × 500 cGy never happened in history. The case is a type I anomaly, where the prescription itself has not been seen before historically.
- WBRT is generally given for patients with multiple brain mets at a dose of 300 cGy in 10 fractions. However, WBRT is also offered to a subset of patients with small-cell lung cancer to reduce the probability of spread of tumor to the brain (This is called PCI). The actual RT field is identical, but the dose is slightly different. For the PCI indication, the dose is 250 cGy × 10 fractions. It can be easy to inadvertently prescribe 300 cGy × 10 fractions for the PCI indication. From the review perspective, the plans are identical and the site name for both is WBRT. This is an example of a type II anomaly.
- Esophageal cancer can be treated with a prescription of 180 cGy × 25 fractions delivered once daily to 4500 cGy. Small cell lung cancer is usually treated with 150 cGy × 30 fractions delivered twice daily to a total dose of 4500 cGy. An accidental prescription of 180 cGy × 25 fractions (BID) to 45 Gy for a small cell lung cancer patient has occurred at least once in clinical practice. According to testimony from a radiation oncologist, it was not easily caught because 180 cGy and 4500 cGy are seen so often, even though it was incorrect. It was caught midway through treatment when the patient was having more toxicity than expected (probability because they were receiving a higher dose per fractions for a BID treatment than they should have been). This is an example of a common prescription being mixed into an incorrect group of patients or a type II anomaly.

These examples serve to illustrate the types of things that can go wrong in clinical practice and are the target of the tool. Note that the example cases above come from testimony (the clinical experience) of physicians, not from an analysis of prescriptions in the historical database. For a discussion of the possibility of anomalies in the historical database and how it relates to our model and pipeline, please see Supplementary Notes.

### The distance model

The model defines a logical system that will flag the new patient if its ‘distance’ from other patients in the historical database or specific groups of patients in the historical database is too large. In order to compare the new patient’s prescription and other features with patients in the historical database, we need to define some pairwise and group-level dissimilarity metrics. For this reason, we have defined two such distance metrics: a *prescription distance* to indicate the distance in the prescription parameters, and a *feature distance* to indicate the distance within the remaining features included in the model.

The pairwise prescription distance, *ρ*_*Rx*_(*i*, *j*) between the new patient, *i*, to any historical patient, *j*, in the database, is simply the Euclidean distance of the scaled prescription features,

1 $${\rho }_{Rx}(i,j)=\sqrt{{({\widetilde{f}}^{i}-{\widetilde{f}}^{j})}^{2}+{({\widetilde{d}}^{i}-{\widetilde{d}}^{j})}^{2}}$$

where $\widetilde{f}$ and $\widetilde{d}$ is the min-max scaled fractions *f* and dose per fraction *d*.

The pairwise feature distance, *g*_*F*_(*i*, *j*), between the new patient, *i*, and any historical patient, *j*, in the database, is the *Gower* distance calculated overall features that are NOT prescription-related. The Gower distance^27^ provides a simple way of computing dissimilarity when mixed numerical and categorical features are present. Numerical features contribute based on the absolute value of the difference divided by the range. In contrast, the dissimilarity is one for categorical features if they are different and zero if they are the same. Each feature in the Gower distance is given equal weight so that the Gower metric has a range on the interval [0,1].

In addition to pairwise dissimilarity metrics, we also define the "closest-m group distance” of the new patient *i*, *R*(*i*), defined as the average of the *m* shortest prescription distances between patient *i* and patient’s *j* in the historical data.

2 $$R(i,m)=\frac{1}{m}\mathop{\sum}\limits_{j\in m-closest}{\rho }_{Rx}(i,j)$$

Similarly, we also define a “closest-*n* group distance”, *F*(*i*), for all non-prescription-related features that apply the same formula but summing over *n* pairwise Gower distances between the new patient, *i* and patients, *k*, in the historical database. We restrict the sum to patients *k* who have either *the same prescription* as patient *i* or who have minimal prescription distance to patient *i*. For example, if *n* = 10 and there are 12 patients with the same prescription as patient *i* in the historical, we select the lowest 10 Gower distances from this group of 12. If *n* = 20, then first we would include all 12 terms *ρ*_*Rx*_(*i*, *k*) = 0 in the sum to compute *F* and then sort over the next closest prescription distance to find remaining terms similarly. We choose this metric because we expect features to be more similar when compared to others with the same (or similar) prescription.

3 $$\begin{array}{l}F(i,n)=\frac{1}{n}\mathop{\sum}\limits_{k\in n-closest}{g}_{F}(i,k) \\ {{{\rm{where}}}}\,n\,{{{\rm{terms}}}}\,{{{\rm{determined}}}}\,{{{\rm{by}}}}\,{{{\rm{sorting}}}}\,{{{\rm{by}}}}\,{\rho }_{Rx}(i,j)\,{{{\rm{then}}}}\,{{{\rm{by}}}}\,{g}_{F}(i,k)\end{array}$$

In order to define thresholds that will define our cutoff for flagging, it is helpful to calculate some characteristic values of pairwise distances in the historical dataset. In this way, we can precisely define what we mean when we say two patients’ features are similar or dissimilar. We can say they are dissimilar if their feature distance is much larger than the average historical pairwise distances for two patients with the same prescription. We compute the mean pairwise prescription distance and the mean pairwise feature distance over all pairs of patients in the historical database to get a typical distance, *θ* and *τ*, defined by

4 $$\theta =\frac{1}{S(S-1)}\mathop{\sum}\limits_{j,k}{\rho }_{Rx}(j,k)$$

5 $$\tau =\frac{1}{S(S-1)}\mathop{\sum}\limits_{j,k}{g}_{F}(j,k)$$

where *S* is the number of patients in the historical database and, again, *ρ*_*Rx*_(*j*, *k*), *g*_*F*_(*j*, *k*) are distances between a pair of historical patients *j* and *k*.

Then, we pattern the thresholds as percentages of these characteristic values as follows:

6 $${t}_{Rx}=a\theta$$

where *a* is a model parameter to be determined by optimization. If *R* > *t*_*Rx*_ then we flag it as an anomaly (type I).

Similarly, we define the feature threshold as a ratio of some characteristic values such as

7 $${t}_{F}=b\tau$$

where *b* is a model parameter. If *F* > *t*_*F*_ then we flag as anomaly (type II).

**Fig. 4:**
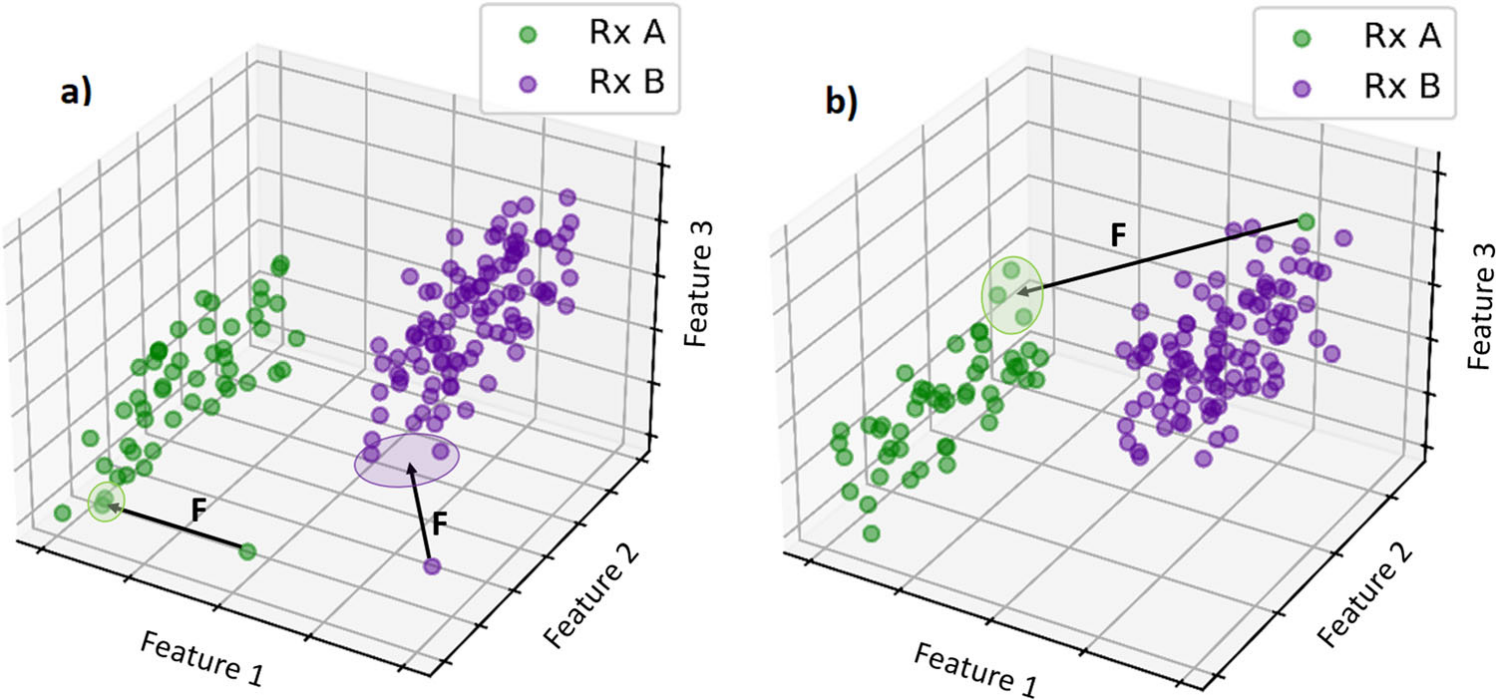
Illustration of different anomalous cases the model is designed to catch. In **a**, we show two feature anomalies that are far from the average. In **b** a case with prescription A is mismatched within the feature sector of prescription B.

In Fig. 4, two different feature anomaly scenarios are depicted in a purely illustrative 3D feature space. In both cases, anomalies can be detected if far away from the *n*-group centroids belonging to their prescription. Note that in the diagram, the *n*-group centroids are determined by the data points on the surface of the prescription cluster closest to each anomaly data point. In Fig. 4, panel a), the anomalies are isolated in the feature space, whereas in Fig. 4, panel b) a single anomaly is mismatched into an incorrect prescription sector of the feature space.

**Fig. 5:**
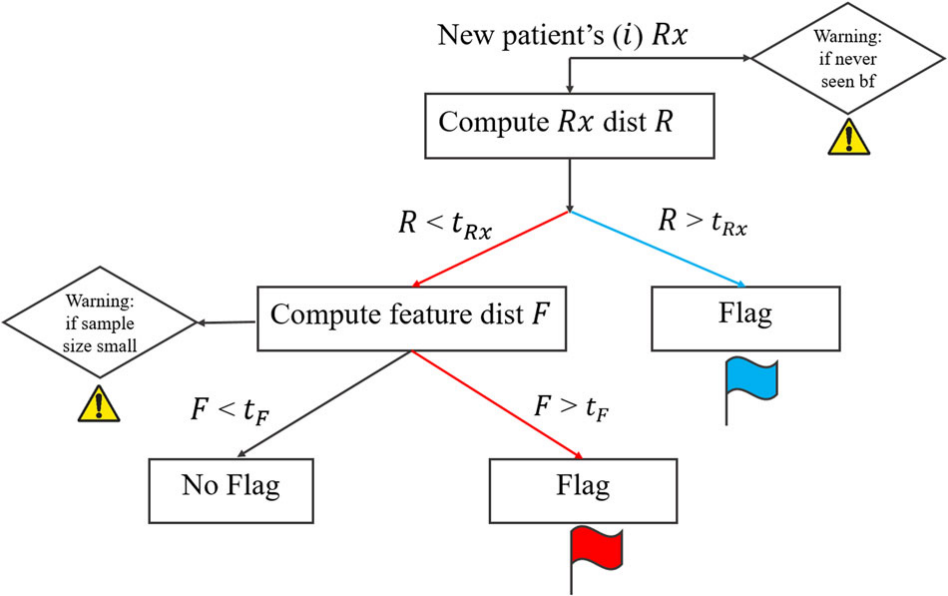
Model architecture. We use dissimilarity metrics *R* and *F* to flag incoming new patient. If prescription is uncommon (*R* is greater than *t*_*Rx*_), we flag it as blue. Otherwise, we compute feature distance *F*, if it is greater than cut off *t*_*F*_, we flag it as red indicates that feature mismatches with its prescription. We can also give warnings as shown.

The logic of the model is depicted in the decision tree shown in Fig. 5. The first step is to compute the closest-*m* prescription group distance, *R*(*m*), and flag if it is larger than some threshold *t*_*Rx*_. If *R* is too large, then the new patient’s prescription is too dissimilar when considering other prescriptions in the historical database. If *R* < *t*_*Rx*_ then we compute the closest-*n* group feature distance considering only patients with the same prescription as patient *i* in the *F* calculation. A warning is given if there are no *n* patients in the historical database with the same prescription as the new patient, *i*. If *F* is more than some threshold *t*_*F*_, we flag the new patient for the mismatch between the prescription and their other features, at least for the data in the historical database. The model was implemented in python.

### Model training

We have four parameters in this model: *m*, *n*, *a*, *b*. In order to scale with the size of the historical dataset, the parameters *m*, *n*, are re-expressed as percentages of the historical training set size. Thus *m* = *μS*, where *S* is the number of samples in the historical database per technique after subtracting a holdout set, and *μ* is the parameter we use for hyper optimization. Similarly, we define *n* = *νS* and optimize of over the percentage *ν*. Thus our final set of parameters for optimization are *μ*, *ν*, *a* and *b*.

We used a parameter space search (grid-search) optimization to determine these parameters. The objective function for optimization was taken as the *f*1 score ($f1=\frac{{t}_{p}}{{t}_{p}+\frac{1}{2}({t}_{p}+{f}_{n})}$; *t*_*p*_ is true-positives and *f*_*n*_ is false-negatives) over a training set that includes 10-30 simulated anomalies (SAs) and a similar number of non-anomalous patients. Thus, the training set consists of SAs and holdout data from the historical database so that we have both positive (anomaly) and negative (not anomaly) classes in the test set.

Optimization through parameter space search was implemented with python *hyperopt* module^28^. Hyperopt uses the tree Parzen Estimator (TPE) to search the parameter space efficiently. Search intervals were defined based on the characteristic values *θ* and *τ* for parameters *a* and *b*. Search intervals for the percentages *μ* and *ν* were constrained to be between 0 and 0.1, which confines the *m*, *n*-group dissimilarity metrics to 10% of the historical database or lower for calculations of *F* and *R*. The number of evaluations was set to 100 per each space search of the detection algorithm.

In order to reduce variance in the normal (not-anomaly) class, we averaged the results over random samplings of the non-anomalous holdout historical records. During this averaging, the anomaly class data points remained constant because we had a limited number of simulated anomalies available for training. This process was demonstrated in Supplementary Figure 2. For easy navigation, we provide a notation summary in Supplementary Table 5.

### Synthesization of anomalies based on distribution

Creation of the anomalies is a time-consuming task that requires careful examination of the historical database and identification of non-previously-occurring patterns between prescription and other features. We will illustrate the construction with some examples below. The main idea is to change the prescription of an existing record, or to change the other features of an existing record, in a way that creates a data point that is not typical of historical prescription-feature patterns. In this way we create *a mismatch* between the prescription and the other features. This mismatch is verified by observing conditional distributions of features based on the given prescription for each case. Thus we carefully check that the anomalies constructed are rare based on the historical conditional distributions.

We must construct simulated anomalies that would be similar to those that could occur in the actual setting. We can obtain the correct parameters to generalize the model’s application to the real world by carefully designing the anomalies. We expect to tune the model parameters to catch each of the simulated anomalies and flag them.

Simulated anomalies were generated by switching the leading digit in the fractions with the leading digit in the dose per fraction or by varying several feature values randomly so that the resulting features do not match the prescription. In Table 3, we show four examples, marked A - D, where the original record is placed above its anomalous mutated form. In example A, we switched the fractions (Fx) and dose per fraction (Dose/Fx) from 5 *fx* x 400 *cGy* to 4 *fx* x 500 *cGy*. 5 *fx* x 400 *cGy* is a common prescription in 3D thoracic treatment, having occurred 50 times in the historical database but not 4 *fx* x 500 *cGy*, which occurred only once.

**Table 3:** Simulated anomaly examples.

Example		Fx	Dose/Fx (*cGy*)	Age at Tx	Technique	Energy (*MeV*)	Intent	ICD10 code	Morphology code
A	orig	5	400	76	3D	mixed photon	–	C34.90	80463
	mutate	4	500	76	3D	mixed photon	–	C34.90	80463
B	orig	5	1000	91	SBRT	6fff	curative	R91.1	–
	mutate	5	1000	10	SBRT	6fff	palliative	R91.1	–
C	orig	4	1200	49	SBRT	6	palliative	C34.30	87203
	mutate	4	1200	49	SBRT	10	palliative	C15.9	87203
D	orig	10	300	74	3D	15	palliative	C78.1	–
	mutate	10	300	74	IMRT	15	palliative	C78.1	–

– stands for missing values.

The simulated anomalies were created in B and C by modifying other features and leaving the original prescription intact. For example, we changed the treatment intent from curative to palliative in case B and the age from 91 to 10. The prescription 5 *fx* x 1000 *cGy* occurred 185 times in SBRT thoracic treatment but never occurred with palliative intent. Also, this prescription was never used in a pediatric patient (age under 21). Thus we varied the features in a way that created a mismatch between prescription and diagnostic features. In C, we mutated the diagnostic code from C34.30 to C15.9. Compared with the historical records, this prescription never treated the esophagus (which has a diagnostic code in the C15 series) and only was used to treat the lungs (C34 series). Also, we mutated the energy from 6 MeV to 10 MeV, which never occurred for this prescription.

In the last example, D, we simulated an anomaly by switching the technique label from 3D to IMRT so that effectively all the features are mismatched. 10 *fx* x 300 *cGy* is a common prescription in both 3D and IMRT. The feature sets are pretty distinct because in 3D, the energy that comes with this prescription is usually 15 MeV, but 15 MeV rarely occurs in historical IMRT cases.

It should be noted that this approach to simulating anomalies is purely data-driven and based on deviations from past historical patterns. The anomaly creation process was done by authors with no clinical information (MDs were excluded from this process).

### Reporting summary

Further information on research design is available in the Nature Research Reporting Summary linked to this article.

## Supplementary information

Supplementary Material

Reporting Summary

## Data Availability

Data is not available due to ethical/legal restrictions by the current IRB.

## Code availability

Our code is available for public here: https://github.com/qli622/Rx_anomaly_detection.git.
