# Supplementary material for: A digital physician peer to automatically detect erroneous prescriptions in radiotherapy
Source: NPJ Digit Med. 2022 Oct 21;5:158. doi: 10.1038/s41746-022-00703-9 (PMC9586941; doi:10.1038/s41746-022-00703-9)
Supplement: Supplementary file 1 — Supplementary Material [file 41746_2022_703_MOESM1_ESM.pdf]

SUPPLEMENTARY INFORMATION

Supplementary Table 1. Raw Features. IDA and IDE refer to JHU institution specific ID tags.

**Features**
IDA
IDE
Last Name
First Name
Birth of Date
Physician Signature Date
Revised Physician Signature Date
Attending
Site Name
Fractions
Dose per Fraction
Total Dose
Total Dose Accumulation
Technique
Energy
Tx Intent
Morphology Code
Description of Morphology Code
Diagnostic Code
Description of Diagnostic Code
T Stage
N Stage
M Stage
Stage Group
Diagnosis Addendum
Tumor Marker (ER)
Tumor Marker (PgR)
Tumor Marker (Her2)
Gleason Score (Primary)
Gleason Score (Secondary)
Gleason Score (Tertiary)
Tx Frequency

Supplementary Table 2. Diagnosis codes included in the thoracic model.

**Diagnosis code (ICD10)**	**Description of the code**
C15.3	Malignant neoplasm of upper third of esophagus
C15.4	Malignant neoplasm of middle third of esophagus
C15.5	Malignant neoplasm of lower third of esophagus
C15.9	Malignant neoplasm of esophagus, unspecified
C33	Malignant neoplasm of trachea
C34.00	Malignant neoplasm of unspecified main bronchus
C34.01	Malignant neoplasm of right main bronchus
C34.02	Malignant neoplasm of left main bronchus
C34.10	Malignant neoplasm of upper lobe, unsp bronchus or lung
C34.12	Malignant neoplasm of upper lobe, left bronchus or lung
C34.2	Malignant neoplasm of middle lobe, bronchus or lung
C34.30	Malignant neoplasm of lower lobe, unsp bronchus or lung
C34.31	Malignant neoplasm of lower lobe, right bronchus or lung
C34.32	Malignant neoplasm of lower lobe, left bronchus or lung
C34.80	Malignant neoplasm of ovrlp sites of unsp bronchus and lung
C34.81	Malignant neoplasm of ovrlp sites of right bronchus and lung
C34.82	Malignant neoplasm of ovrlp sites of left bronchus and lung
C34.90	Malignant neoplasm of unsp part of unsp bronchus or lung
C34.91	Malignant neoplasm of unsp part of right bronchus or lung
C34.92	Malignant neoplasm of unsp part of left bronchus or lung
C37	Malignant neoplasm of thymus
C38.1	Malignant neoplasm of anterior mediastinum
C38.2	Malignant neoplasm of posterior mediastinum
C38.3	Malignant neoplasm of mediastinum, part unspecified
C38.4	Malignant neoplasm of pleura
C38.8	Malignant neoplasm of ovrlp sites of heart, mediastinum and pleura
C45.0	Mesothelioma of pleura
C77.1	Secondary and unsp malignant neoplasm of intrathorac nodes
C78.00	Secondary malignant neoplasm of unspecified lung
C78.01	Secondary malignant neoplasm of right lung
C78.02	Secondary malignant neoplasm of left lung
C78.1	Secondary malignant neoplasm of mediastinum
C78.2	Secondary malignant neoplasm of pleura
D15.0	Benign neoplasm of thymus
E85.8	Other amyloidosis
R91.1	Solitary pulmonary nodule

Supplementary Table 3. Energy used in different techniques

**Energy**	**3D**	**IMRT**	**SBRT**
x06	293	1896	277
x06FFF	0	71	615
x10	77	56	1
x10FFF	0	1	0
x15	521	94	8
Mix Photon	144	76	1
Mix Mode	1	0	0

Supplementary Table 4. Data row example for distance model input

Rx-related features		Non-Rx-related features					
Fx	Dose/fx(cGy)	Age at Tx	Technique	Energy	Intent	ICD10 code	Morphology code
4	1200	63	SBRT	x06	palliative	C34.10	81406

Supplementary Table 5. Model parameters notation

**Notation**	**Definition**
ρ_{Rx}	pairwise prescription distance (scaled euclidean)
g_F	pairwise feature distance (gower distance)
R	closest-m group average prescription distance
F	closest-n group average gower distance
S	number of patients in the historical data base
θ	mean pairwise prescription distance
τ	mean pairwise feature distance
m	parameter controlling number of patients to average over for R statistic
n	parameter controlling number of patients to average over for F statistic
μ	m expressed as percentage of historical database
ν	n expressed as percentage of historical database
t_F	threshold for flagging on F statistic
t_{Rx}	threshold for flagging R statistic
a	optimization parameter for setting t_F threshold
b	optimization parameter for setting t_{Rx} threshold
s_n	number of normal samples is train / testing set
s_a	number of anomalous samples in train / testing set
f_1	binary classification: $f_1 = \frac{t_p}{t_p+\frac{1}{2}(t_p+f_n)}$; t_p true-positives, f_n false-negatives

Supplementary Table 6. Mock peer review examples (performance of physicians and model)

<table>
<tr><th colspan="11">Case 1</th></tr>
<tr><td rowspan="2"></td><th>Tx site</th><th>Fx</th><th>Dose/Fx(cGy)</th><th>Tech.</th><th>Energy</th><th>Tx intent</th><th>ICD10 code</th><th>Morph. code</th><th>Age at Tx</th><th>Truth</th></tr>
<tr><td>lul nodule</td><td>10</td><td>5000</td><td>SBRT</td><td>x6fff</td><td>curative</td><td>R91.1</td><td>-</td><td>73</td><td>Anomaly</td></tr>
<tr><td>MD1</td><td>Flag</td><td colspan="9">“Dose/Fx error, unsafe”</td></tr>
<tr><td>MD2</td><td>No Flag</td><td colspan="9"></td></tr>
<tr><td>MD3</td><td>Flag</td><td colspan="9">“Prescription error”</td></tr>
<tr><td>Model</td><td>Flag</td><td colspan="9">Type I anomaly. R = 1.982, t_{Rx}=0.002</td></tr>
<tr><th colspan="11">Case 2</th></tr>
<tr><td rowspan="2"></td><th>Tx site</th><th>Fx</th><th>Dose/Fx(cGy)</th><th>Tech.</th><th>Energy</th><th>Tx intent</th><th>ICD10 code</th><th>Morph. code</th><th>Age at Tx</th><th>Truth</th></tr>
<tr><td>lul centr nodule</td><td>5</td><td>1000</td><td>SBRT</td><td>x6fff</td><td>palliative</td><td>R91.1</td><td>-</td><td>10</td><td>Anomaly</td></tr>
<tr><td>MD1</td><td>No Flag</td><td colspan="9"></td></tr>
<tr><td>MD2</td><td>Flag</td><td colspan="9">“palliative not usually used to define lung SBRT;
age atypical for lung SBRT”</td></tr>
<tr><td>MD3</td><td>Flag</td><td colspan="9">“seems odd for 10 yr-old to receive such dose,
but I don’t know peds cases very well.”</td></tr>
<tr><td>Model</td><td>Flag</td><td colspan="9">Type II anomaly. F = 0.269, t_F = 0.234</td></tr>
<tr><th colspan="11">Case 3</th></tr>
<tr><td rowspan="2"></td><th>Tx site</th><th>Fx</th><th>Dose/Fx(cGy)</th><th>Tech.</th><th>Energy</th><th>Tx intent</th><th>ICD10 code</th><th>Morph. code</th><th>Age at Tx</th><th>Truth</th></tr>
<tr><td>left airways</td><td>10</td><td>300</td><td>IMRT</td><td>x15</td><td>palliative</td><td>C78.1</td><td>-</td><td>74</td><td>Anomaly</td></tr>
<tr><td>MD1</td><td>Flag</td><td colspan="9">“Why IMRT used for palliative?”</td></tr>
<tr><td>MD2</td><td>Flag</td><td colspan="9">“IMRT for palliative dosing (300/10) is not typical”</td></tr>
<tr><td>MD3</td><td>No Flag</td><td colspan="9"></td></tr>
<tr><td>Model</td><td>Flag</td><td colspan="9">Type II anomaly. F = 0.585, t_F = 0.438</td></tr>
<tr><td colspan="11">- stands for missing values</td></tr>
</table>

In Supplementary Table 6, the first case is a simulated anomaly (SA), where the original record was 5 x 1000. Both MD1 and MD3 identified it as an prescription anomaly, however, MD2 missed. The model predicted it as a type I anomaly because the evaluation metrics R is greater than t_{Rx}.

The second case is also a SA, because we changed the age from 91 to 10 and treatment intent from curative to palliative. The fact that the treatment intent for this prescription is never curative and never been given to a pediatric patient creates a mismatch between the feature set and the prescription, even though the prescription is one of the most popular prescription in historical SBRT database. MD2 seems to found both atypical properties of this patient while MD3 found one reason and MD1 missed it completely. The model predicted it as a type II anomaly because the evaluation metrics F is greater than t_F.

The last case is also a SA, where we change the label from 3D to IMRT, that all of the features became mismatched with this prescription. MD1 and MD2 identified it as anomaly but MD3 missed it. The model predicted as a type II anomaly because again F is greater than t_F.

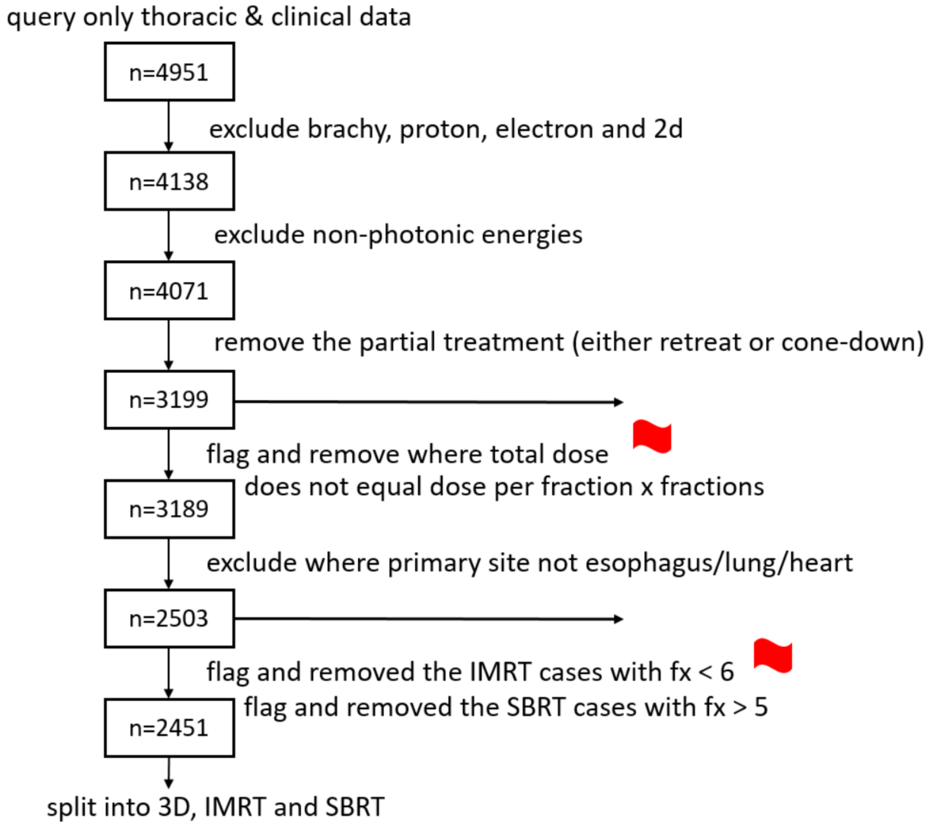

Supplementary Figure 1. **Consort diagram tracking the number of patients at each pre-processing step**. n is the number of records remaining at each step. The red flag indicates where in the pipeline has the flagging checkpoint.

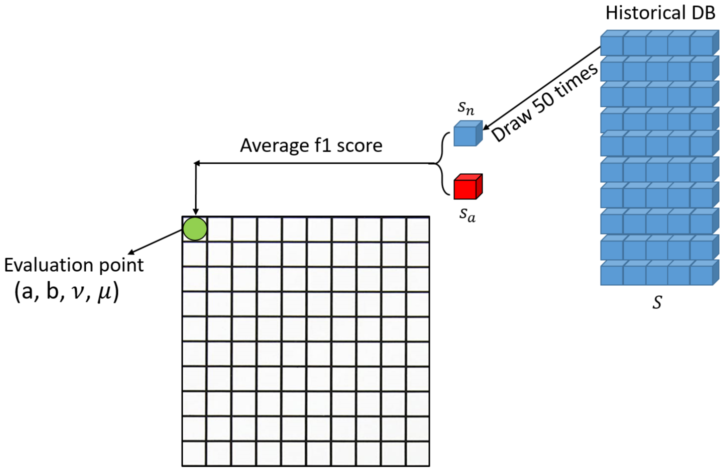

Supplementary Figure 2. **An illustration of the training process**. In each point in parameter grid space (a, b, ν, μ), we run the model 50 times, where for each run, normal samples (size of s_n) are randomly chosen from the historical database (size of S). In the meanwhile, anomaly samples (size of s_a) are kept the same. After the 50 runs, we will average $f1$ score and record the value for this evaluation point. Then, we move on to the next evaluation point. Eventually, 100 grid points are evaluated. Among those, we chose the parameter set that will give the highest $f1$ score. It is possible that this optimized parameter set is not unique (two grid points can provide the same highest $f1$ score).

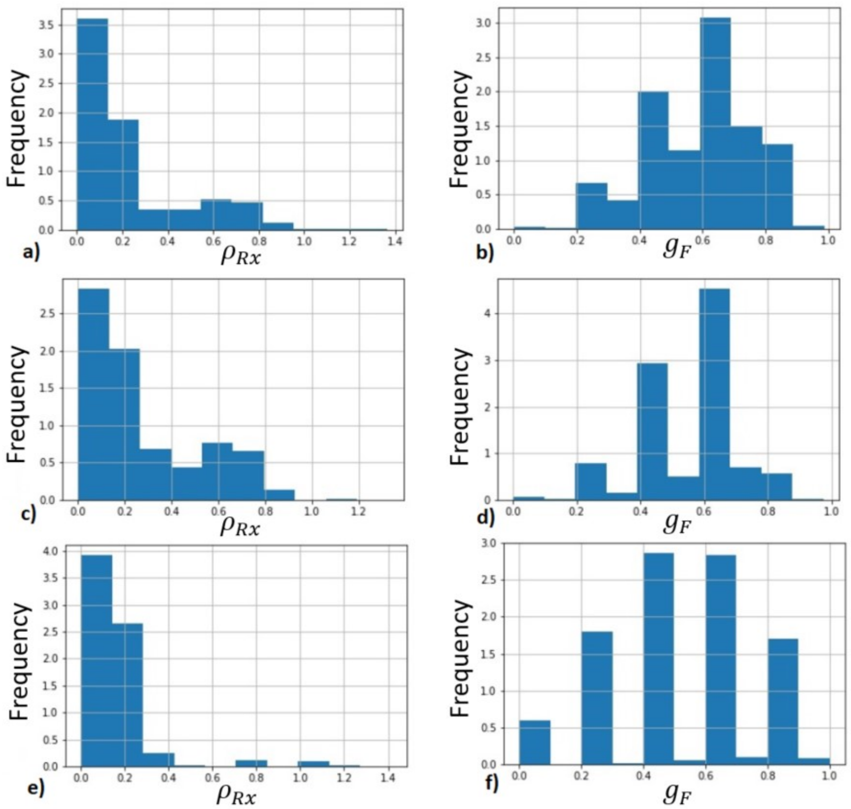

Supplementary Figure 3. **Normalized histograms of prescription and feature distances in the historical patients' database.** Panel **a)-b)** 3d **c)-d)** IMRT **e)-f)** SBRT. Left panel is the distribution of pairwise prescription distance $\rho_{Rx}(j, k)$ and right panel is distribution of Gower feature distance $g_F(j, k)$ with j, k are historical patients' pair. Spikes relate to the three categorical features through the dice similarity. If one categorical feature is different the net dissimilarity is 0.2, if two categoricals are different it is 0.4 etc. In SBRT, the pairwise Gower distances are dominated by categorical features.

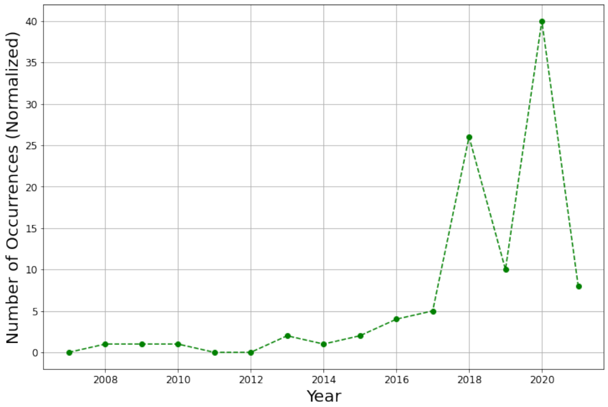

Supplementary Figure 4. **Case study shows data reflecting protocol changes**. The plot is number of occurrences of hypofractionated prescriptions (300cGy, 350cGy and 400 cGy in 15 fractions) grouped for each year normalized by the number of total patients treated that year (percentage). We observe a take-off in 2016. The publication which generated this trend in the field was in September of 2015 [1].

Supplementary Notes

Secular trends in prescription variables

Detection of secular trends, or changes over time in the prescriptions is a relevant question to our study, and thus we analyzed our historical database to see if we can detect such trends. One reason they are relevant is because it might be the case that a prescription (treatment guideline) was very popular in the past but has become outdated by today's standards. In that case, we would not want our historical database, and anomaly flagging pipeline to be indifferent to shifting trends in Rx and dosing over time. Such indifference could lead our model to the wrong conclusions due to the insensitivity to secular trends.

Our approach was to calculate the yearly prevalence of each prescription over the time span of our data (2007-2020). By prevalence we mean the fraction of thoracic patients that were prescribed a specific dose divided by the total number of thoracic patients that year. We then conducted linear regression of the prevalence against time controlling to some extent confounding variables such as yearly variation in the patients average age following the methodology of a previous large study of secular trends in treatment [2].

We conducted the secular trend analysis on 2424 cases from the thoracic historical database spanning the time period between 2007-2020. Our analysis is divided by RT technique, IMRT ($n = 1172$), SBRT ($n = 723$) or 3DCRT ($n = 529$). We only retained those data points wherein the prescription occurred at least 20 times in the historical database. The remaining data are show in Supplementary Table 8. We found that for IMRT there were 12 prescriptions that occurred more than 20 times in the historical database, but for SBRT and 3DCRT only three prescriptions met this criteria.

For the following analysis we split the data into three parts by treatment technique, analyzing the IMRT, SBRT and 3DCRT subsets separately. The prevalence of the prescription, meaning the number of times that prescription occurred within a given year, divided by the total number of cases for that year is shown in Supplementary Table 7 for the sample prescription *28*x*180* along with the average age of all the patient's from that year. Additionally, to simplify the linear regression, the years 2007-2020 are mapped to the integer's 0-13. As mentioned we regress prevalence on the mapped time regressor as well as average age.

Linear regression was carried out in python with the *statsmodel* package. Regression p-values for the time coefficients for each prescription are shown in Supplementary Table 8. We found four statistically significant results at the $\alpha = 0.05$ significance level, (without adjusting for multiple comparisons). The prevalence versus year for each of the four prescriptions that showed statistically significant secular trends are plotted in Supplementary Figure 5. We can see that the *25*x*180* prescription was gradually abandoned in IMRT over the time-span, while there was about a 5% increase in the adoption of the *15*x*300* prescription. In SBRT, the *4*x*1200* prescription reduced from 80% prevalence down to below 40% in recent years.

Additionally, secular trends were also detected by calculating the change in average prevalence in the first and last third of the time series. By first and last third we mean the period from 2007 to 2020 was divided into the sub-period 2007-2012 (first third) and 2016-2020 (last third). This method works well to detect cases where there is a sudden

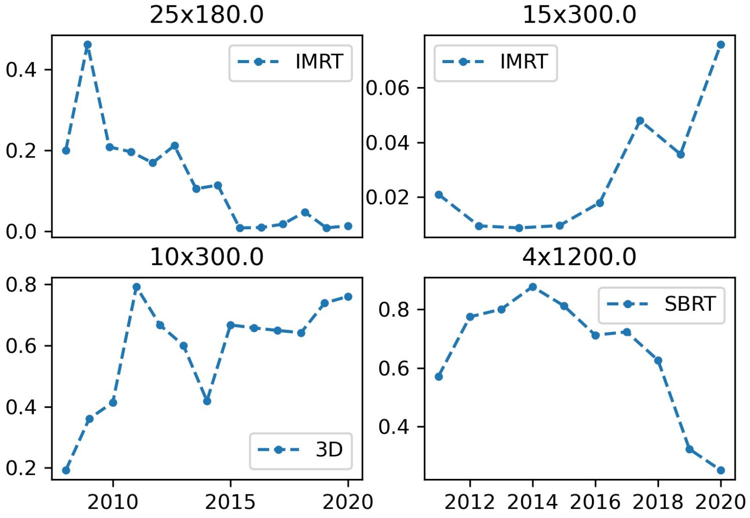

Supplementary Figure 5. Prescriptions that showed statistically significant secular trends by regression method. The vertical axis is the yearly prevalence of the prescription.

Supplementary Table 7. Example of yearly prevalence prescription data for IMRT prescription *28x180*

year	Age_Tx	year_mapped	prevalence
2007	66	0.0	0.200
2008	65	1.0	0.038
2009	55	2.0	0.167
2010	58	3.0	0.054
2011	67	4.0	0.123
2012	67	5.0	0.106
2013	66	6.0	0.105
2014	64	7.0	0.095
2015	69	8.0	0.263
2016	66	9.0	0.202
2017	68	10.0	0.189
2018	63	11.0	0.200
2019	66	12.0	0.268
2020	66	13.0	0.117

non-linear adoption or abandonment of a RT prescription instead of a gradual increases or gradual decreases which are well detected by linear regressions. Choosing a tolerance of 10% change in prevalence between first third and last third time-sections of the historical database, we detected secular trends where the average prevalence change is more than this tolerance. The secular trends detected with this second method agreed with the results from the linear regression though it failed to catch the IMRT *10*x*300* since the change was less than 10%. It did successfully detect the other three cases detected by linear regression and no other cases were detected by this method.

For this analysis, we define the RT "prescription" as the combination of both the fractions and the dose per fraction. However, a more complete analysis of prescription data should look for secular trends in other prescriptive features

Supplementary Table 8. Number of occurrences of prescriptions in the historical database (thoracic) split by technique. We only considered those prescriptions with at least 20 observation in historical database. Also shown are the regression p-values of the time coefficient for the regression.

**Prescription**	**IMRT**	**3D**	**SBRT**	p
28x180	190	–	–	0.210
33x200	185	–	–	0.771
30x200	98	–	–	0.239
35x180	94	–	–	0.302
25x180	93	–	–	**0.00015**
34x180	77	–	–	0.788
30x150	59	–	–	0.203
30x180	53	–	–	0.375
15x400	46	–	–	0.997
32x200	42	–	–	0.269
15x300	28	–	–	**0.034**
10x300	21	317	–	0.994,**0.015**
5x400	–	50	–	0.709
10x250	–	23	–	0.331
4x1200	–	–	417	**0.0127**
5x1000	–	–	185	0.689
4x1250	–	–	75	0.641

such as Total dose, Biologically effective dose as well as to consider fractionation and dose per fraction separately as treatment variables. We intend to expand the secular trend detection component of the pipeline to search through a comprehensive set of prescription treatment variables in the future.

It should be noted that there is a limitation in the secular trend detection in that con-founders are not controlled for. In the linear regression detection method, we were able to control for average age in the year's cohort of patients, though we did not control for disease structure (e.g. aggregate yearly differences in diagnostic code and disease severity makeup of patient population across years). A more thorough analysis would create several aggregate variables to encode variation in aggregate disease structure from year to year.

Our main result is that we were are able to detect statistically significant secular trends in the historical database. Depending on the sign of the time coefficient we can impose a warning when this prescription is encountered to notify the physicians that this prescription has been increasingly adopted or abandoned over time. Going forward, in the next iteration of our model, we do plan to incorporate both of the secular trend detection methods as part of our pipeline. Specifically, early in the pipeline, we will conduct the secular trend detection over the historical database and then provide the described warning if detection occurs.

Discussion and Analysis of historical database anomalies and comparison to synthetic

To aid the following discussion and analysis of the role of anomalies with respect to the historical data we begin by giving several more specific definitions of what we mean by “anomalies”:

- **Data artifact anomalies -** These refer to anomalies that occur due to problems with data management. For example, a test record not associated with any real patient is introduced into the database but not removed after testing. Also, flagrant typos in the data that occur after a successful correct treatment. In these cases, we define them by the condition that no real patient was harmed but the clinical data was not captured correctly or did not persist correctly in the database.

- **Real anomalies -** This refers to a case where the wrong prescription was delivered (an accident) or was nearly delivered (a near-miss) to the patient due to human errors. Patient was likely harmed or came close to being harmed. These could be type I or II anomalies.

- **Simulated anomalies -** This refers to imaginary cases of Real anomalies that are constructed based on clinical knowledge (e.g. the mistakes that are more likely to happen).

Our approach was to remove all of the data artifact anomalies as well as to attempt to flag a number of obviously suspicious cases based on knowledge of certain standard prescription guidelines that are rarely breached. Together these form the “***Pre-processing*** **anomalies**”. In this *pre-processing* step of the historical database we do not claim to have been exhaustive, as we cannot scrutinize every non-standard case in the historical database because that would be impractical. The key point is that what was previously referred to as “real anomalies” is only a subset of the possible real anomalies.

When our tool flags a type I or II anomaly, it is serving as a warning – a call to closer examine the case. There may be ambiguity in that the flag could be a false positive. Recall that to train the distance model, we use the historical database as the reference “non-anomalous” class. This is an essential component of our model, and assumes that the historical database is error free. When training the model, we tune model parameters using different objective functions depending on the desired false-positive rate. We need to point out that we often tend to choose a high false-positive rate since the cost of false-negative is much greater than the cost of creating a false-positive.

Therefore, if we were to try to reevaluate some historical cases with some trained version of our distance model, the many possible flagging events would make it impractical to do manual investigations to determine if each flagging event is a true-positive or a false-positive. Our model is intended to be forward looking. It is not intended to be used to look backward into the historical database. Again, a case-by-case clinical investigation of each flagged historical case in the past is impractical.

It is possible that subtle type II or type I errors may exist in our historical database, however, they are very unlikely since the historical database reflects post peer review data. In practice, when a mistake in prescription is found, the prescription would be corrected. In order for the error to exist in the historical database it would have to have never been caught at any point by the patient’s care team. Still, if such cases exist, we do not think it would hurt the

predictive ability of our model, since our model creates warnings for rare events, and the existence of a few misclassfied cases in the past wouldn't change the fact that they are rare. (See the m and n group dissimilarity functions which can reduce the effect of any single data point in the historical database.)

In order to clean the historical database of these *pre-processing anomalies* (as defined above), our approach was to manually inspect our raw historical data and remove anomalies. The criteria for manual filtering was based on applying logical rules that check for nonsensical doses, highly atypical energies for a given technique, or for highly abnormal fractions or dose per fraction for a given treatment technique. We found that for IMRT there were 30 cases where the number of fractions was inconsistent with practice and 2 cases where the total dose did not equal the number of fractions times the dose per fraction, yet other clinical features seemed normal. In particular, we chose to label any IMRT case where the number of fractions was less than six as a historical database anomaly. For 3DCRT we found four cases where the total dose did not equal the number of fractions times the dose per fraction. For SBRT, we found 8 cases where the energy was inconsistent with clinical practice and 9 cases where the dose fractionation did not meet normal clinical standards. We removed SBRT cases where the number of fractions was more than 5. This data is summarized in Supplementary Table 9.

As we mention in the Discussion section, just because we flag something as an historical anomaly does not necessarily mean that the wrong prescription (dose) was delivered to the patient, however it may be sufficiently unusual that it would warrant further scrutiny, even if it turns out to be a false positive.

This component in the pipeline requires manual inspection of historical data and we do not have an automated systematic solution for it because it depends on the quality of the queried institutional historical data. The rules we imposed are based on clinical knowledge of typical guidelines and institutional patterns for RT prescription.

Here we perform an analysis on the anomalies that were removed from the raw historical database as described in section 2.3. We test the performance of the distance model on these historical *pre-processing* anomalies and see if there are any differences between these anomalies and the synthetic anomalies that were created in section 2.4.3. It is important to emphasize that these *pre-processing* anomalies are just a subset of the "real-world anomalies" and do not reflect all possibilities as described above.

Supplementary Table 9 gives the number of anomalies removed from the historical database broken down by both technique as well as the reason why it was flagged. These *pre-processing* anomalies were input into our distance model following the same methods as described for the synthetic anomalies in Results Section. The results are shown in Supplementary Table 11. s_a refers to the number of *pre-processing* anomalies fed into the distance model for each technique, whereas s_n refers to the number of normal historical samples tested alongside the anomalies. As before, to reduce variance from false positives, we repeated for 50 different samplings of the historical normal inputs and then computed the error bar in the f1 score from the sample standard deviation. We chose to equally balance anomalous and normal samples. The other parameters in the table are the same as in the results section, except for *tpr* which refers to the true positive rate in the binary classification.

Supplementary Table 9. Number of preprocessing anomalies broken down by technique and reason for removal

	Technique	Total dose $\neq$ fx $\times$ dose per fx	Non-standard fx	Non-standard energy
Historical DB Anomalies	3D	4	–	–
	IMRT	2	30	–
	SBRT	–	9	8

Model parameters were chosen from the previous optimizations over the synthetic training set. For IMRT, the best performance was found by using the parameters that were optimized over the Rx switching task. This is due to the nature of the IMRT *pre-processing* anomalies primarily being caused by strange prescriptions that are very far outside normal guidelines so that the R value is large. For IMRT, we obtained a 0.95 f1 score with perfect recall (true-positive rate) on the *pre-processing* and the only incorrect predictions were false positives on the normal class. Thus we can say that the distance model was easily able to flag the *pre-processing* IMRT anomalies.

Supplementary Table 10. Parameters and model performance scores.

	Technique	a	b	ν	μ	τ	θ	$f1$	tpr	s_n	s_a	S
Historical DB Anomalies	3D	0.010	0.707	0.010	0.037	0.581	0.206	0.85 $\pm$ 0.08	1.0	4	4	509
	IMRT	0.265	0.979	0.025	0.014	0.543	0.261	0.95 $\pm$ 0.02	1.0	32	32	1153
	SBRT	1.926	0.465	0.010	0.075	0.501	0.142	0.70 $\pm$ 0.03	0.65	17	17	704

Results for 3D treatment technique were similar to IMRT though with only four *pre-processing* anomalies it is difficult to draw strong conclusion, however, for 3D the *tpr* was also a perfect 1.0 indicating that these anomalies were easy to detect due to the large R values. For SBRT, the distance model performed perfectly on the *pre-processing* l anomalies that were flagged because dose fractionation did not meet SBRT criteria of being 5 or less fraction however, it was only able to correctly detect approximately 50% of the SBRT *pre-processing* anomalies that were withheld due to the rare energy of the treatment.

Supplementary Table 11. Average m-group prescription distance, $\bar{R}$ for *pre-processing* and simulated anomalies broken down by RT technique. The *pre-processing* anomalies were taken from the historical database, the simulated anomalies are the ones from the out of sample testing.

	historical DB anomalies	simulated anomalies
3D	0.23	0.137
IMRT	0.85	0.138
SBRT	2.38	2.039

In our model construction there are two condition wherein we flag an input, either if $R > t_R$ or if $F > t_F$. These two conditions are exhaustive in the sense that all possible anomalies should fit the condition that either the prescription is atypical or other features are atypical. With that in mind, when we look for aggregate differences between the anomalies from the historical database, and the simulated anomalies that we constructed, we will not find any qualitatively different types of anomalies, however we may find that the characteristic values such as the mean $\bar{R}$ or $\bar{F}$ values are quantitatively different.

In Supplementary Table 11 we see that the average $\bar{R}$ is indeed higher for the *pre-processing* anomalies than it is

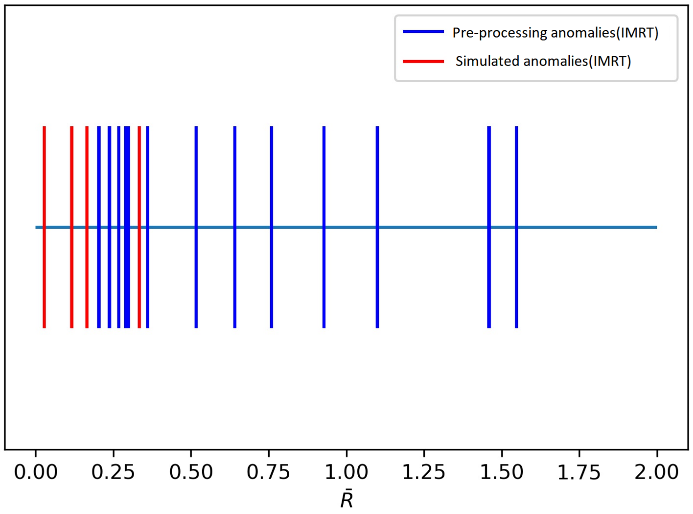

Supplementary Figure 6. One dimensional plot of IMRT prescription m-group distance $\bar{R}$ for simulated anomalies in red and historical database anomalies (subset) in blue. The simulated anomalies are from the out of sample test set.

for the simulated ones (the average is over simulated anomalies from the out sample test set). When we created the simulated anomalies, we wanted to have more subtle variations in the prescription than the sometimes flagrantly non-standard anomalous prescriptions that were discovered in the historical database, in some cases. This is also shown in Supplementary Figure 6 which shows a one dimensional plot of the individual values of $\bar{R}$ for the *pre-processing* anomalies in blue and the simulated anomalies in red (there are some overlap of the red data points which explains why only 4 bars are visible).

It is important to remember that the historical database *pre-processing* anomalies analyzed only represent a subset of possible real anomalies. This subset, in our data, tended toward conspicuous prescription anomalies with large R values, which makes sense because they were selected for being obviously (flagrantly) violating clinical standards. One conclusion is that it makes sense to use simulated anomalies in order to capture more subtle error possibilities.

Comparison to Supervised Learning

To get some sense of the relative performance of our model versus standard machine learning models, we trained and tested random forest models with the exact same inputs as we fed to the distance model. The in-sample (training) dataset consisted of the entire historical dataset for each technique plus the same anomalies used to train the distance model's parameters. The class imbalance in the in-sample data was handled by random minority oversampling so that classes were balanced.

However, due to the repetitions of the same data in the minority class, cross-validation is no longer a viable option to select hyper-parameters. Cross-validation does not work since the anomalies present across all k-folds are the same. Since we do not have enough anomaly records to form both validation and test sets, the hyperparameters of the RF were determined by performance on the test set. We optimized the number of estimators (trees) and the maximum depth of each tree. Furthermore, due to variance induced by the random oversampling (ROS), we averaged in-sample results over 100 ROS runs. An example of the typical in-sample over-fitting curve / out-sample optimum is shown in Supplementary Figure 7 where the f1 score is plotted vs. hyperparameter maximum depth for a fixed number of 20 estimators per forest.

Supplementary Table 12. Parameters and model performance for random forest compared to distance model

	d	n-trees	RF in-sample f1 (averaged)	RF out-sample f1	distance model out-sample f1
**3D**	3	10	0.86	0.82	0.941
**IMRT**	3	10	0.84	0.52	0.727
**SBRT**	3	10	0.9	0.77	0.875

The out-of-sample results refer to the same test set on which we ran the distance model. Results for the RF are shown in Supplementary Table 12. We can see that the RF performs significantly worse than our distance model. It should be noted that the RF models we constructed could potentially be optimized better; we do not wish to suggest that our model necessarily outperforms all supervised learning models, but rather that it clearly outperforms simple supervised learning models such as RF optimized over two hyperparameters. Certainly, more work could be done on the supervised learning side to improve the supervised learning results. Nevertheless, the out-performance of the distance model compared to the supervised learning approach above shows that the distance model may be a superior anomaly detection tool.

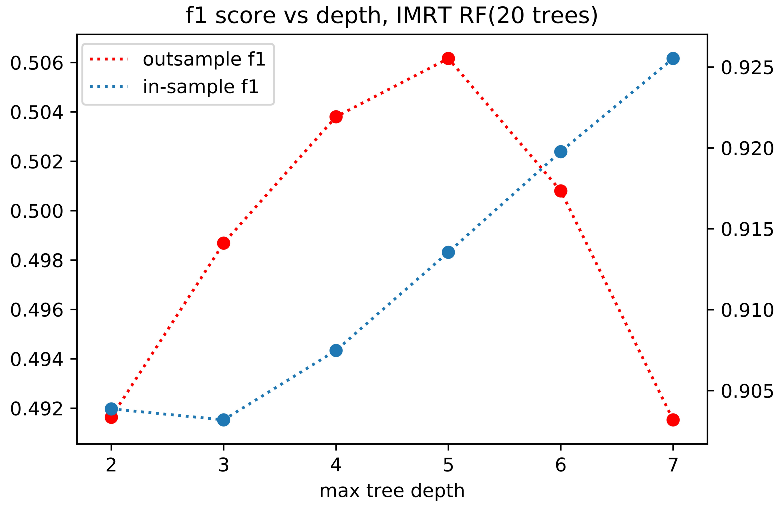

Supplementary Figure 7. Typical over-fitting / optimum curves for hyperparameter optimization. The vertical scale on the right belongs to the in-sample, whereas the left vertical axis pertains to the out-sample curve. Optimization is performed over the test set whereas, ideally, hyperparameter optimization should be conducted on a validation set or with cross-validation (CV). Because CV does not work here, we do not have sufficient anomalies to form validation and test sets.

SUPPLEMENTARY REFERENCES

[1] Kenneth D Westover, Billy W Loo Jr, David E Gerber, Puneeth Iyengar, Hak Choy, Maximilian Diehn, Randy Hughes, Joan Schiller, Jonathan Dowell, Zabi Wardak, et al. Precision hypofractionated radiation therapy in poor performing patients with non-small cell lung cancer: phase 1 dose escalation trial. *Int. J. Radiat. Oncol. Biol. Phys.*, 93:72–81, 2015.

[2] Douglas Redd, Yijun Shao, Yan Cheng, and Qing Zeng-Treitler. Detecting secular trends in clinical treatment through temporal analysis. *J. Med. Syst.*, 43(3):1–7, 2019.
